# Raised Source/Drain (RSD) and Vertical Lightly Doped Drain (LDD) Poly-Si Thin-Film Transistor

**DOI:** 10.3390/membranes11020103

**Published:** 2021-02-01

**Authors:** Feng-Tso Chien, Jing Ye, Wei-Cheng Yen, Chii-Wen Chen, Cheng-Li Lin, Yao-Tsung Tsai

**Affiliations:** 1 Department of Electronic Engineering, Feng Chia University, Taichung 407, Taiwan; m0821871@o365.fcu.edu.tw (J.Y.); m0829658@o365.fcu.edu.tw (W.-C.Y.); cllin@fcu.edu.tw (C.-L.L.); 2 Department of Electronic Engineering, Minghsin University of Science and Technology, Hsinchu 304, Taiwan; cwchen@must.edu.tw; 3 Department of Electronical Engineering, National Central University, Taoyuan 320, Taiwan; tsai@ee.ncu.edu.tw

**Keywords:** raised source/drain (RSD), lightly doped drain (LDD), thin film transistor (TFT), kink effect

## Abstract

The raised source/drain (RSD) structure is one of thin film transistor designs that is often used to improve device characteristics. Many studies have mentioned that the high impact ionization rate occurring at a drain side can be reduced, owing to a raised source/drain area that can disperse the drain electric field. In this study, we will discuss how the electric field at the drain side of an RSD device is reduced by a vertical lightly doped drain (LDD) scheme rather than a RSD structure. We used different raised source/drain forms to simulate the drain side electric field for each device, as well as their output characteristics, using Integrated Systems Engineering (ISE-TCAD) simulators. Different source and drain thicknesses and doping profiles were applied to verify the RSD mechanism. We found that the electric fields of a traditional device and uniform doping RSD structures are almost the same (~2.9 × 10^5^ V/cm). The maximum drain electric field could be reduced to ~2 × 10^5^ V/cm if a vertical lightly doped drain RSD scheme was adopted. A pure raised source/drain structure did not benefit the device characteristics if a vertical lightly doped drain design was not included in the raised source/drain areas.

## 1. Introduction

Recently, polycrystalline silicon thin-film transistors (Poly-Si TFTs) with low temperature processes fabricated on plastic or glass have been widely used in memory, active-matrix liquid crystal display (AMLCD), peripheral driver and pixel switches circuits, and 3-D integrated circuits, because of their high field effect mobility and driving current compared with amorphous silicon [1,2]. The conventional poly-Si TFT has a high electric field near the channel/drain junction. It is the major cause of impact ionization, which leads to a serious kink effect and degrades the device characteristics and breakdown voltage [3,4]. This drawback also limits poly-Si TFTs to be used in digital and analog circuit on glass for high resolution displays [5].

In order to reduce this high drain electric field, many structures have been proposed to overcome this problem. Offset [6,7], lightly doped drain (LDD) [8], gate overlapped lightly doped drain (GOLDD) [9], and raised source/drain (RSD) [10,11,12] are some of the proposed schemes that can effectively reduce the drain side electric field. Conventional RSD devices contain thick source/drain (S/D) regions and a thin channel, are not self-aligned in nature, and need additional masks [13]. A self-aligned RSD structure needs additional chemical mechanical polishing (CMP) processes. The works in the literature [12,14] proposed an RSD device combined with an offset gated structure, that also employed a double-channel to improve the high series resistance in the offset region and increased the on-state current. A double-gate RSD scheme was also proposed to enlarge the on-current performance [10,15]. The characteristics of all of these RSD structures are considered to be improved by raised source/drain (RSD) regions that can spread the high horizontal electric field at the drain area [16].

**Figure 1:**
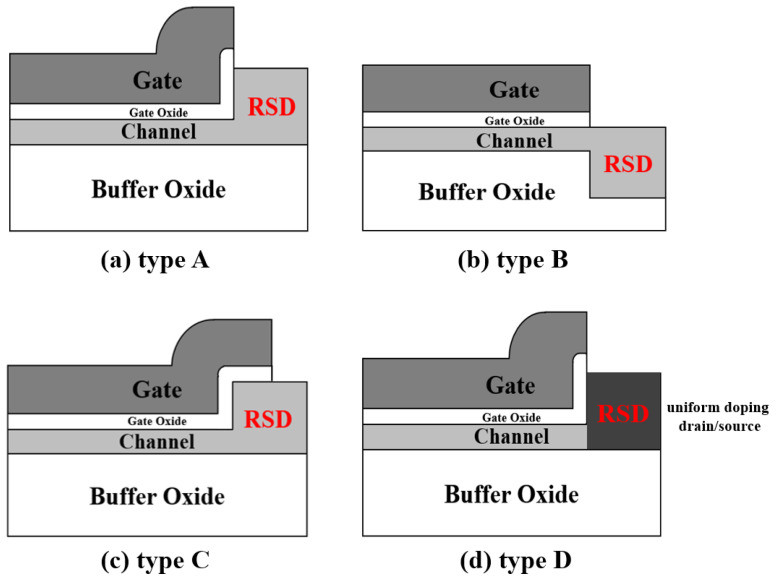
The cross section for various types of raised source/drain (RSD) structures.

In this study, we found that a thick drain/source area could not reduce the drain electric field of a TFT device. The reason for the lower drain side electric field is that the thick drain region formed a non-uniform doping profile in the drain area, which could be thought of as a vertical lightly doped drain structure. We designed four different RSD structures and compared their drain electric field under the same RSD thicknesses. In order to investigate and compare the drain electric field and drain doping profile in all of the designed TFT frames, 2D numerical simulations were carried out using the Integrated Systems Engineering (ISE-TCAD) device simulator [4,17]. The four different RSD structures are shown in Figure 1. Various source/drain thicknesses and doping concentration profiles for the devices (as shown in Figure 1) were adopted in order to investigate the device drain side electric field. We also fabricated a type-C device, which was the easiest structure to implement and confirm our model.

## 2. Devices Design

In the past, the drain breakdown mechanism in ultra-thin-film silicon-on-insulator (SOI) MOSFET’s has been explained, and given that a high electric field can be dispersed by increasing the thickness of the drain terminal, it can thus improve the breakdown voltage [18]. This might be true for the same drain/channel thickness structures, where the drain doping curvature will affect the drain side electric field; however, this is not the case for an RSD scheme. Here, we constructed four different RSD structures (shown in Figure 1). All of the devices shown in Figure 1 have a thicker drain/source region than the channel thickness. The drain/source region for all of the structures was implemented by phosphorous implantation (dose = 5 × 10^15^ cm^−2^ with 45 keV), and then activated at 600 °C for 8 h in a N_2_ ambient.

**Figure 2:**
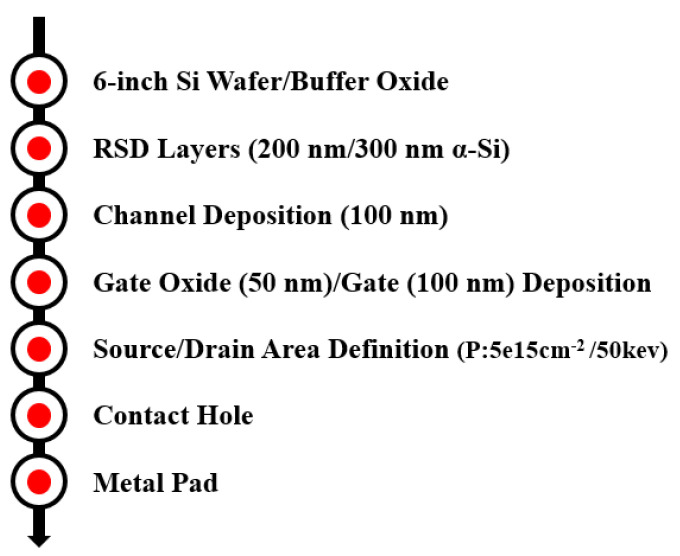
The simulation flow diagram for the ISE-TCAD device simulator.

**Figure 3:**
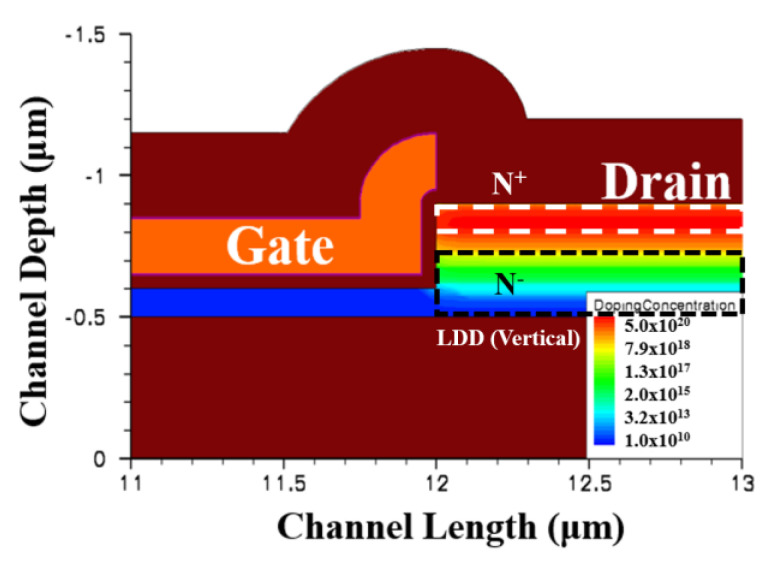
The simulated drain region doping concentration of a type-A RSD structure.

Figure 2 illustrates the device simulation flow diagram using an ISE-TCAD device simulator. The design and process parameters for the simulation were identical to those used in the implementation. Figure 3 shows the simulated drain region doping concentration of a type A RSD structure plotted using an ISE-TCAD device simulator. Because the drain region was much thicker than the channel, the vertical doping concentration demonstrated a lightly doped drain (LDD) scheme after the drain/source annealing process. We predicted that this vertical LDD structure plays an important role in lowering the drain side electric field. In order to verify our prediction, we also used a uniform doping concentration in the drain/source regions for the device shown in Figure 1a in order to investigate its drain electric field. All of the simulated deices had the same dimensions, with a gate length of 10 μm. The channel thickness for all of the structure was 100 nm. In this study, we used three different RSD thicknesses (200 nm, 300 nm, and 400 nm) to analyze the electric field distributions for various raised source/drain films.

**Figure 4:**
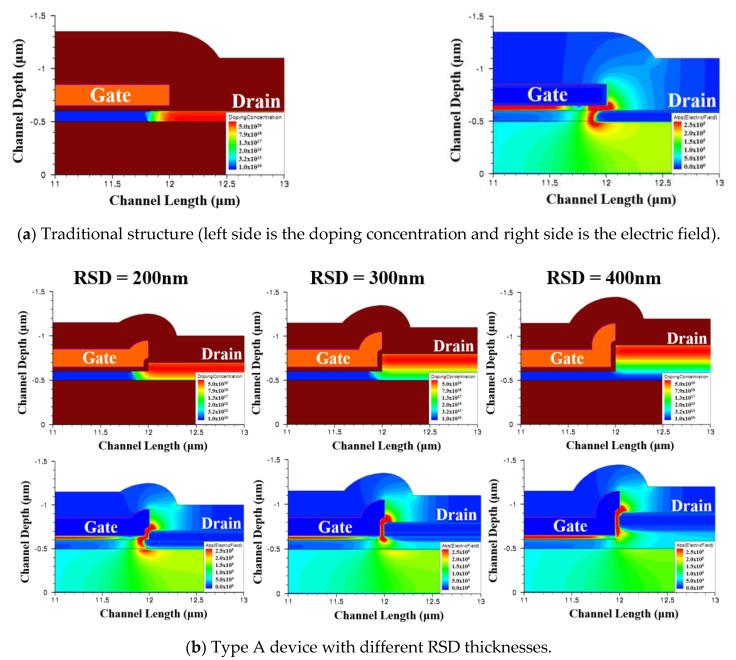

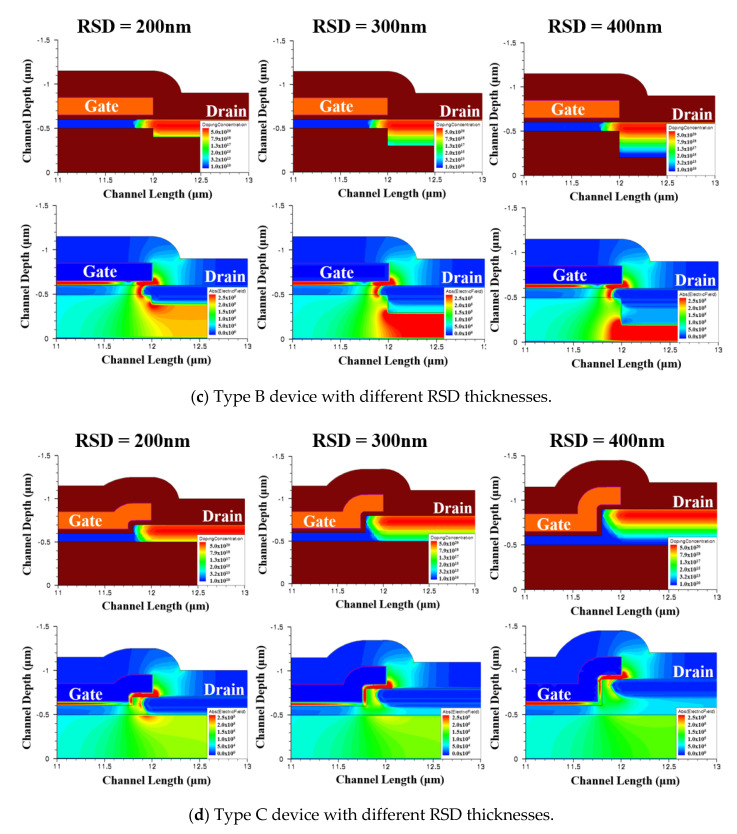

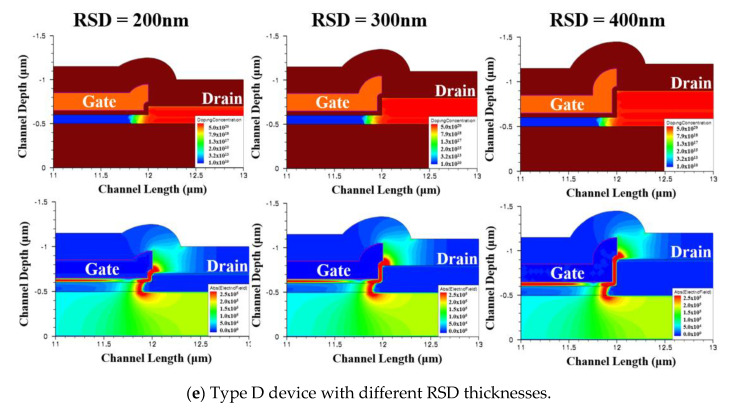
Drain side doping concentration profile (above) and drain electric field distributions (below) for a (**a**) traditional thin-film transistor (TFT) and (**b**-**e**) different types of RSD TFTs with RSD thicknesses of 200 nm, 300 nm, and 400 nm.

To analyze the TFT drain side electric field distributions and doping profiles for all of the RSD structures, an ISE device and process simulator was carried out for the device analysis [19]. The simulated device structures and characteristics could be adequately simulated by taking into consideration the presence of the spatially uniform density of state (DOS), the conventional drift-diffusion model, and the local impact ionization model (where we used the Chynoweth model [4,20]) [9,10]. Four exponential distributions, two tail states (donor- and acceptor-like tail states) and two deep level states (donor- and acceptor-like deep state states), were sufficient to describe the DOS in the poly-Si devices [21]. For the sake of simplicity, the ISE-TCAD default material parameters were applied to simulate the electric fields for all of the structures. The electron ionization coefficients and DOS at the donor-like states in an n-type device play more import roles than the hole and acceptor-like ones. The characteristic energy of the exponential for the donor-like tail state and the peak of the exponential for the donor tail state parameters used in the simulations were 35 (100) meV and 1 × 10^21^ (5 × 10^18^) cm^−3^eV^−1^, respectively. These values for the donor-like deep state and donor tail deep state were 100 meV and 5 × 10^18^ cm^−3^eV^−1^, respectively. The capture cross section for the donor and acceptor states were 1 × 10^−14^ cm^2^ and 1 × 10^−16^ cm^2^ for electrons, while those values were 1 × 10^−16^ cm^2^ and 1 × 10^−14^ cm^2^ for the holes, respectively. Figure 4a shows the simulated doping profiles and electric field distributions for a traditional poly-Si TFT device after the drain/source region implantation annealing process, and Figure 4b–d illustrates the same characteristics for all of the structures mentioned in Figure 1 with different drain/source thicknesses near the channel/drain region under the same process. Figure 4e shows the performance of the type A structure with a uniform doping drain/source region. The above parts of Figure 4b–e are the doping concentration profiles, while the ones below are the corresponding drain side electric field distributions. The electric field of all figures were extracted at V_GS_ = 5 V and V_DS_ = 8 V. The simulated maximum drain electric field of a traditional TFT is 2.98 × 10^5^ V/cm. These values for the type A, B, C, and D structures with an RSD thickness of 200 nm (300 nm and 400 nm) were 2.85 × 10^5^ V/cm (2.56 × 10^5^ V/cm and 2.05 × 10^5^ V/cm), 2.96 × 10^5^ V/cm (2.88 × 10^5^ V/cm and 2.85 × 10^5^ V/cm), 2.64 × 10^5^ V/cm (2.21 × 10^5^ V/cm and 2.01 × 10^5^ V/cm), and 2.98 × 10^5^ V/cm (2.97 × 10^5^ V/cm and 2.97 × 10^5^ V/cm), respectively.

From Figure 4, it is obvious that all of the RSD structures led to a vertical LDD structure, except the uniform doping drain/source one; however, the current route for the different RSD structures affected whether the current passed the LDD area or not. Even though the type-B device was an RSD structure, its current path did not pass the vertical LDD area. This meant that the RSD design of type B device was not beneficial for reducing the drain electric field. We found that no matter how thick the drain area we designed in the type B device, the current of all TFTs always flowed through the channel to a heavy doped drain. In addition, the drain electric field of the type B TFTs almost all remained the same as the one shown in the traditional device. This implied that a raised source/drain region was not the reason for the improvement of the device’s characteristics. For type A and type C devices, the current could flow through a vertical LDD area, therefore reducing the drain electric field. We observed that the thicker drain/source region of the type A or type C structure had a longer vertical lightly doped drain distance, which helped to lower its drain electric field. The distinction between the type D and type A devices was the drain/source region doping conditions. The type A device was a vertical LDD structure through drain/source annealing, while the type D design was a uniform doping drain/source area device. We also observed that the current of the type D device did not go through an LDD area, which indicates that the drain electric field was almost as high as the one shown in the traditional TFT.

**Figure 5:**
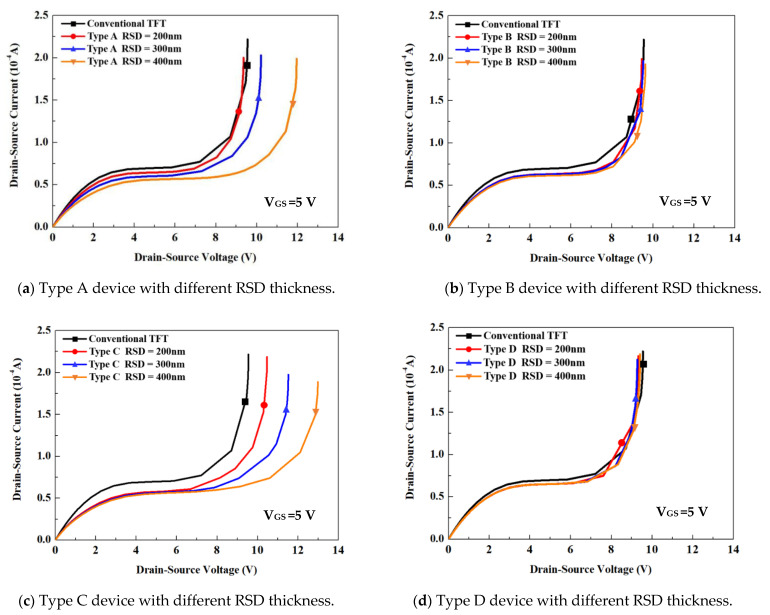
(**a**–**d**) The I_DS_-V_DS_ characteristic curve comparison for all of the RSD structures.

Figure 5 demonstrates the corresponding output characteristics of the structures shown in Figure 4. Note that the emphasis of this study was on the investigation of the physical mechanisms on the RSD structure in a thin-film transistor, rather than on a precise determination of all of the model parameters for a particular batch of devices. Thus, the simulation parameters we used in [9] were thought to be sufficiently accurate. The output performance for all of the drain/source thicknesses of type B and type D devices had almost the same I_DS_-V_DS_ characteristics as the convention one, because the electron current of those TFTs all flowed along the channel to a heavy doped drain area. We observed that the kink effect and breakdown performance of type A and type C devices could be improved owing to the vertical lightly doped drain area formed by the source/drain annealing process. A thicker RSD film led to a longer vertical lightly doped drain path that could effectively lower the drain side electric field. The reduced on-current in the type C device was not as obvious as that in type A, because most of the electron current in the type C device flowed along the channel to the drain area, while most of the electron current in the type A device flowed directly into the LDD area. From Figure 5, it is clear that the RSD structure is not the reason for the improvement in the device performance—the vertical LDD structure that occurred in the raised source/drain area was the cause that improved the device characteristics.

## 3. Device Structure and Fabrications

**Figure 6:**
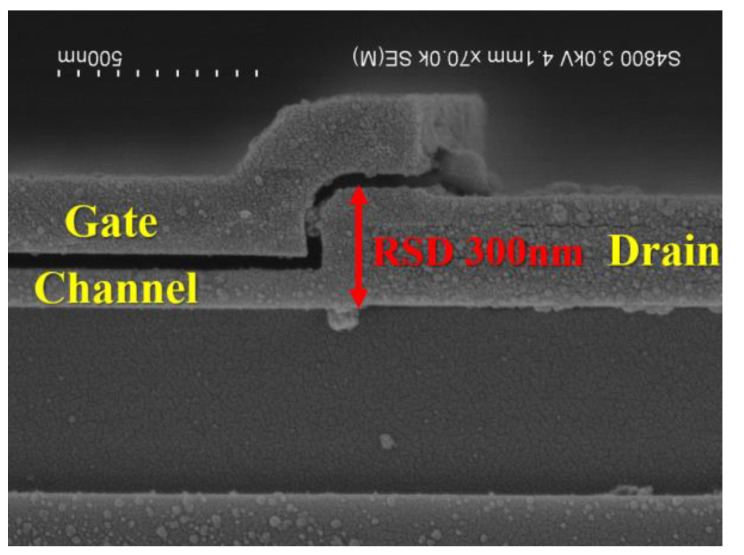
The SEM images of the Type C device with an RSD of 300 nm.

In order to further verify our simulation results, we also fabricated the type C structure, which was the easiest structure to implement and confirm our model. Two different RSD thickness with a uniform doping drain (UDD) and LDD structure were designed and implemented. To fabricate the devices, an in-situ doped poly-Si with two different thickness conditions (100 nm and 200 nm) was first deposited, and defined the RSD area on oxidized silicon wafers to be the uniform doping RSD TFT. For the vertical LDD structure design, the in-situ doped poly-Si was replaced with undoped amorphous silicon (*α*-Si). Then, 100 nm undoped *α*-Si was used as the channel and the layer above the patterned RSD area. After that, the *α*-Si layer was transferred into a poly-Si by solid-phase crystallization (SPC) process, which can be found in our previous report [12]. After that, the gate oxide, poly-si gate, source/drain implantation, passivation, and contact metal processes were the same as those in the literature [12]. Figure 6 shows the scanning electron microscope (SEM) image of the type C device with an RSD thickness of 300 nm.

**Figure 7:**
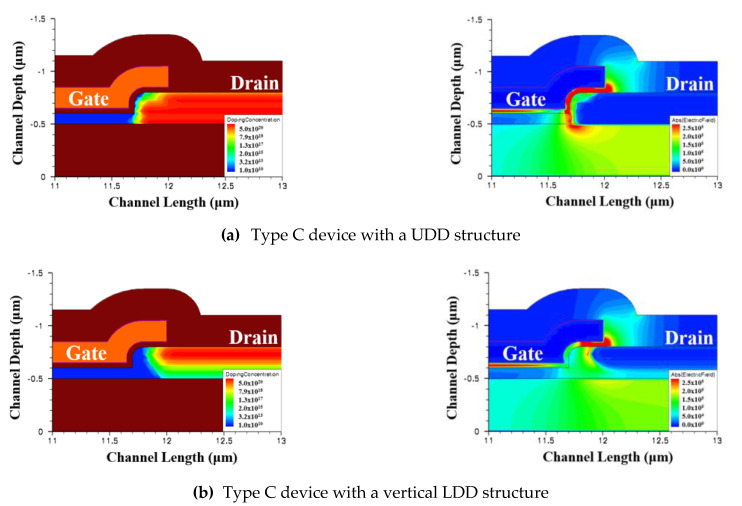
Drain doping concentration profiles and electric field distributions of type C device with uniform doping drain (UDD) (**a**) and vertical lightly doped drain (LDD) structures (**b**) (V_GS_ = 5 V and V_DS_ = 8 V).

Further investigation of the type C device (RSD = 300 nm) with a vertical LDD and UDD through simulation is demonstrated in Figure 7. Figure 7a,b shows the simulated doping profiles and electric field distributions for the type C (RSD = 300 nm) device with vertical LDD and UDD frames, respectively. It is obvious that the electric field was also not reduced for the type C UDD structure. We predict that the output characteristics of the type C device with a UDD design would be the same as those in the tradition device, even if it is an RSD structure.

## 4. Results and Discussion

**Figure 8:**
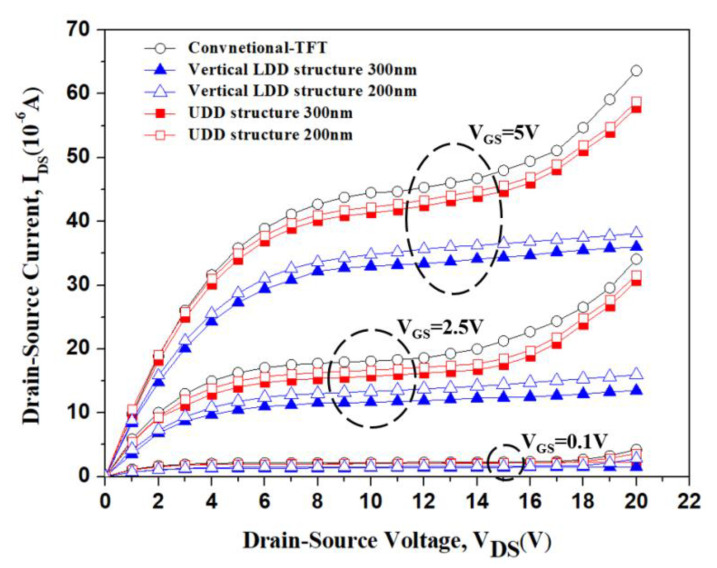
I_DS_-V_DS_ characteristic curves of conventional TFT and type C devices with vertical LDD and UDD.

Figure 8 shows the measured I_DS_-V_DS_ characteristics of conventional TFT and type C devices with vertical LDD and UDD. Over 30 devices for each design were measured within one wafer. The I_DS_-V_DS_ characteristics were almost the same for the identical structure. All of the devices with different fabrication conditions were implemented under the same run in order to reduce the wafer lot to lot vibration. From Figure 8, we can see that the UDD devices with 200 nm and 300 nm RSD structures did not alleviate the kink effect, because the electron flow of these devices went through the channel directly to a heavy doped drain. Even though the UDD devices were RSD structures, the RSD frame did not provide any advantages to improve device characteristics. On the contrary, the type C device with a vertical LDD design achieved an effective improvement on the output characteristics. The thicker undoped RSD thickness following a drain implantation process led to a longer vertical LDD length that was be beneficial to lower the drain electric field, and improved the device breakdown voltage. The on-state current of all of the vertical LDD structures was lower than that of the UDD and conventional devices, because of parasitic resistance [9,22]. The on-current of the type C device with a thick vertical LDD was almost the same as the one with a thin vertical LDD, because most of the electron current of the type C device flowed along the channel to the drain area. The slightly reduced on-current of the type C device with a thick vertical LDD might have been due to the process window which was ±10%, which led to slightly different characteristics between the different wafers. Therefore, the vertical LDD was the main reason for the improved device performance, rather than the RSD frame.

## 5. Conclusions

In this paper, the main reason for the improved device characteristics of the RSD structures was investigated and discussed. Different RSD structures were designed to understand the reason for reducing the device drain electric field by using ISE-TCAD simulators. We found that the electric fields of a traditional device and uniform doping RSD structures were almost the same (~2.9 × 10^5^ V/cm). The maximum drain electric field could be reduced to ~2 × 10^5^ V/cm if a vertical lightly doped drain RSD scheme was adopted. The pure raised source/drain structure did not benefit from the device characteristics if a vertical lightly doped drain design was not included in the raised source/drain areas. Experiment curves also support the simulation results. Uniform doping drain devices with a 200 nm and 300 nm RSD structure did not alleviate the kink effect, because the electron flow of these devices went through the channel directly to a heavy doped drain. This could mean that the vertical lightly doped drain was the main reason for the improved device performance, rather than the raised source/drain frame. The vertical lightly doped drain concept can be adopted in all TFT devices using others materials.
